# Inhibition of NF-*κ*B/IL-33/ST2 Axis Ameliorates Acute Bronchiolitis Induced by Respiratory Syncytial Virus

**DOI:** 10.1155/2021/6625551

**Published:** 2021-08-04

**Authors:** Liwen Zhang, Yu Wan, Liang Ma, Kaihong Xu, Baojin Cheng

**Affiliations:** ^1^Department of Pediatrics, The Second People's Hospital of Changzhou, Affiliated Hospital of Nanjing Medical University, China; ^2^Department of Digestive Disease, The First People's Hospital of Changzhou, the Third Affiliated Hospital of Soochow University, China

## Abstract

**Background/Aim:**

Bronchiolitis is a common acute lower respiratory tract infectious disease in infants. Respiratory syncytial virus (RSV) infection is one of the main causes. Bronchiolitis can lead to a significant increase in the incidence of asthma in young children, but the mechanism of bronchiolitis transforming into asthma is still unclear. The study was aimed at investigating the role of NF-*κ*B/IL-33/ST2 axis on RSV-induced acute bronchiolitis.

**Methods:**

A total of 40 infants diagnosed with acute bronchiolitis infected by RSV, and 20 normal infants were included in this study. BALB/c mice (6-8 weeks old, 20 ± 1.1 g) were used as study models. Enzyme-linked immunosorbent assay (ELISA), quantitative real time PCR, western blot analysis, immunohistochemical staining, and flow cytometry analysis were performed to examine relevant indicators.

**Results:**

IL-33 level was significantly elevated, and Th1/Th2 ratio is imbalance after in infants with acute bronchiolitis. In vivo study, we found that NF-*κ*B/IL-33/ST2 axis is mediated the Th2 cytokine levels and BAL cell number induced by RSV. Acute bronchiolitis induced by RSV in a mouse model is attenuated after inhibition of NF-*κ*B/IL-33/ST2 pathway. Moreover, we also confirmed that macrophages are important sources of IL-33 and are regulated by NF-*κ*B pathway in RSV-induced mice.

**Conclusion:**

We confirmed that inhibition of NF-*κ*B/IL-33/ST2 axis could attenuate acute bronchiolitis by RSV infected. Our findings not only demonstrate the potential role of IL-33 antibody in attenuating RSV-induced lung damage but also provide a new insight into better prevention of RSV-induced asthma by mediating NF-*κ*B/IL-33/ST2 axis.

## 1. Introduction

Asthma is a respiratory disease involving a variety of inflammatory mediators and cytokines, which has been a serious threat to human health [1]. Numerous studies have shown that viral infection can directly invade 75-300 *μ*m bronchioles, causing necrosis and exfoliation of epithelial cells of bronchioles, infiltration of peripheral lymphocytes, glandular hyperplasia, increased mucus secretion, and narrowing or even blockage of the lumen [2, 3]. Repeated or persistent viral infection eventually leads to chronic airway inflammation and airway hyperreflux [4].

Respiratory syncytial virus (RSV), as the main pathogen of bronchiolitis and pneumonia in infants and young children, is one of the main causes of asthma in young patients [5]. Previous reports showed that RSV infection accounts for 80-85% and 75-80% of asthma cases in children and adults, respectively [6, 7]. Another important reason RSV can induce asthma is that it can activate T helper 2 (Th2) cell-related cytokines such as IL-4, IL-5, and IL-10 and break the body's immune balance [8, 9]. Recent studies have found that the treatment of cytokines secreted by Th2 cells can effectively alleviate the symptoms of acute asthma attack, while the reduction of cytokines secreted by Th1 cells does not significantly alleviate the symptoms of asthma [8, 10]. These findings enrich and improve the classic Th1/Th2 imbalance theory in asthma.

IL-33 is a recently discovered proinflammatory cytokine with a variety of biological functions. It belongs to the IL-1 family with a molecular weight of about 1800 and is located on human 9p24.1 chromosome [11]. Current studies have shown that IL-33 not only induces Th0 cells to differentiate into Th2 cells but also promotes the secretion of Th2-related cytokines IL-4, IL-5, IL-6, and IL-10 in vitro and in vivo [12, 13]. In the study of children with asthma, it was found that the proportion of Th2 cells and the level of IL-33 in serum increased significantly, which were positively correlated with the level of autoantibody IgE in vivo [14].

Suppression of tumorigenesis 2 (ST2) receptor can specifically bind to IL-33 in two forms; one is transmodel ST2 (ST2L), mainly expressed on Th2 surface, and the other is soluble ST2, which can compete with ST2L and bind to IL-33, leading to the decrease of IL-33 function [13]. When ST2 is combined with IL-33, it activates the NF-*κ*B signaling pathway and promotes the release of Th2 cytokines such as IL-4, IL-5, and IL-10 [15]. However, the role of IL-33/ST2 and NF-*κ*B in RSV-induced bronchitis is still unclear.

In our present study, we explored the expression of IL-33 in serum of acute bronchiolitis infants and in lung tissues of RSV-induced mice and investigated its role on Th1/Th2 cell ratio in acute bronchiolitis infants and RSV infected mice. Moreover, we demonstrated that NF-*κ*B/IL-33/ST2 axis is involved in the RSV-induced acute bronchiolitis. Our results suggested that IL-33, ST2, and NF-*κ*B can serve as therapeutic targets in the treatment of RSV infected asthma.

## 2. Materials and Methods

### 2.1. Reagents and Antibodies

Respiratory syncytial virus (RSV) was purchased from Hipower Pharmaceutical (Hipower Pharmaceutical, Guangzhou, China). Fetal bovine serum (FBS) and Dulbecco's modified Eagle's medium (DMEM) were obtained from GIBCOBRL (Gibco, CA, USA). Anti-IL-33 antibody and anti-IgG were obtained from Santa Cruz Biotechnology (SantaCruz, CA, USA). Soluble ST2 (sST2) was purchased from KeyGEN (KeyGEN, Nanjing, China). Pyrrolidine dithiocarbamic acid ammonium salt (PDTC) was obtained from Beyotime (Beyotime, Shanghai, China). Antibodies of p50, p65, ST2, and GAPDH were purchased from Cell Signaling Technology (CST, CA, USA). Lipofectamine 2000 and Opti-MEM were purchased from Invitrogen (Carlsbad, CA, USA). Trizol was obtained from Invitrogen (Carlsbad, CA, USA). Western blot detection chemiluminescence reagents were purchased from Thermo Scientific (Thermo Scientific, CA, USA).

### 2.2. Study Subjects

A total of 40 infants from the Second People's Hospital of Changzhou, Affiliated Hospital of Nanjing Medical University between 2016 and 2018 diagnosed with acute bronchiolitis infected by RSV and 20 normal infants were included in this study. The diagnosis of acute bronchiolitis was based on the latest diagnostic guidelines [16]. The venous blood samples of all the selected cases were taken before treatment and used for later experiments. All cases were excluded from congenital heart disease, cardiopulmonary dysplasia, immunodeficiency, and other serious diseases. The present study was approved by the ethics committee of the Second People's Hospital of Changzhou, Affiliate Hospital of Nanjing Medical University (No. SPH1904880). The written informed consent was obtained from all parents of subjects.

### 2.3. Animal Experiments

BALB/c mice (6-8 weeks old, 20 ± 1.1 g) were randomly divided into six groups: normal control (NC), respiratory syncytial virus (RSV), anti-IgG + RSV, anti-IL-13 + RSV, sST2 + RSV, and PDTC+RSV. Protocols for the RSV-induced mouse models were as previously described [17]. The mice in the RSV infection group were anesthetized by intraperitoneal injection of 3% pentobarbital sodium 0.1 ml/kg, followed by nasal drip of RSV with 100 ml 10^6^ PFU on 6 consecutive days, while the mice in the control group were anesthetized by nasal drip of the same dose of saline. The anti-IgG+ RSV, anti-IL-33+ RSV, sST2+ RSV, and PDTC+RSV groups were pretreated with anti-Rabbit IgG antibody and anti-Mouse IL-33 antibody (30 *μ*g/mouse) by intranasal injection 100 *μ*l 2 h, Ribavirin, sST2, and PTDC by intraperitoneal injection 2 h before challenge with RSV on 3 consecutive days.

All the mice were killed and sampled on 3, 5, and 7 days after RSV infected; the present study was approved by the ethics committee of the Second People's Hospital of Changzhou, Affiliate Hospital of Nanjing Medical University (No. CZ0004-1732).

Serum samples were taken from the spleen of mice after execution, and erythrocytes were removed. The cells were suspended in 10 ml of HBSS wash buffer and counted by a blood cell counter. The cell suspension was centrifuged at 4°C for 10 min at 200 g. Then, the supernatant was discarded, and the precipitate was mixed at a ratio of 0.9 ml of MACS buffer per 10^8^ cells. 0.l ml of magnetic beads (CD90) was added per 10 cells and incubate for 15 min at 4°C in order to extract total T cells. After centrifugation and cell suspension at 4°C, 200 g for 10 min, sufficient MACS buffer was added to the precipitate to reach a concentration of 10^8^ cells/ml and mix well. Pass the cells through 30 *μ*m nylon mesh or 40 *μ*m preseparation membrane. Rinse the filter with 0.1 ~ 0.4 ml of MACS buffer. Place the cells into the upper sample channel of autoMACS. Select the possel program so that the labeled cells will elute from the positive lane.

### 2.4. Enzyme-Linked Immunosorbent Assay (ELISA)

The levels of IL-33, IL-4, IL-10, IL-2, INF-*γ*, and ST2 in infant serum were examined by specific ELISA kits (Cloud Clone Corp, Wuhan, China) according to manufacturer's instructions.

### 2.5. Extraction and Detection of Bronchoalveolar Lavage Fluid (BALF)

PBS (1 ml) was injected into the trachea after the separation of surrounding connective tissue and repeated for 3 times, and the rinsed PBS was recovered. Collected lavage fluid for inflammatory cell counting, and stored lavage fluid at -20°C for cell flow cytometry assay detection.

### 2.6. Quantitative Real Time PCR

Total RNA was extracted from lung tissues by using Trizol (Invitrogen). Superscript II (Invitrogen) was used to carry out reverse transcription qualified with 400 ng according to the manufacturer's instructions. For quantitative real time PCR, SYBR Green Master Mix (Roche) and ABI-7900 system were used, and GAPDH functioned as a loading control. Primers of related genes are listed in Table 1.

### 2.7. Western Blot Analysis

Protein samples were prepared, followed with the SDS polyacrylamide gel electrophoresis, and protein was subsequently transferred onto PVDF membranes. For further detection of related genes, the following antibodies were used: I*κ*B, p65, p50, ST2, and GAPDH. Membranes were blocking in 5% BSA for 2 h at room temperature prior to incubation with primary antibody at 4°C overnight. Membranes were washed 10 min for three times in TBST and then cultured with secondary antibody (1 : 5000) (CST, CA, USA) at -20°C for 2 h. Then, membranes were washed 20 min for three times with TBST. The blots were detected with an ECL plus reagent (Thermo Scientific, Waltham, USA).

### 2.8. Immunohistochemical Staining

The lung tissues in different treatment groups were collected separately and were stained with a rabbit monoclonal anti-mouse IL-33, p65, and ST2 antibody overnight at room temperature, washed, then incubated with the secondary Ab (CST, CA, USA) for 2 h, and washed again. The specific detail steps were performed according to DAB substrate kit (Thermo Scientific, Ma, USA).

### 2.9. Flow Cytometry Analysis

Peripheral blood samples and single cell suspension from lung tissue of mice were collected. FITC, anti-CD30, anti-CD49, and CD11 antibodies were added in samples in turn according to the concentration of the instructions. The 100 P1 antibody system fully suspended the cells and incubated on ice for 30 min under the condition of dark. Add 2% FBS and PBS 1 ml/tube, gently whirl, 1500 rpm, 4°C centrifuge for 5 minutes, discard supernatant; repeat this step once. Stored at 2% FBS. PBS overhanging cells were detected and analyzed using the FlowJo software (version 7.6.5).

### 2.10. Statistical Analysis

Continuous variables were shown as “means ± SD.” For multiple comparisons, One-way ANOVA was performed using the SPSS 22.0 software (SPSS, Inc., USA), and *p* value < 0.05 was considered significant statistically.

## 3. Results

### 3.1. IL-33 Level Is Significantly Elevated and Th1/Th2 Ratio Is Imbalance after RSV Infection in Infants

**Figure 1:**
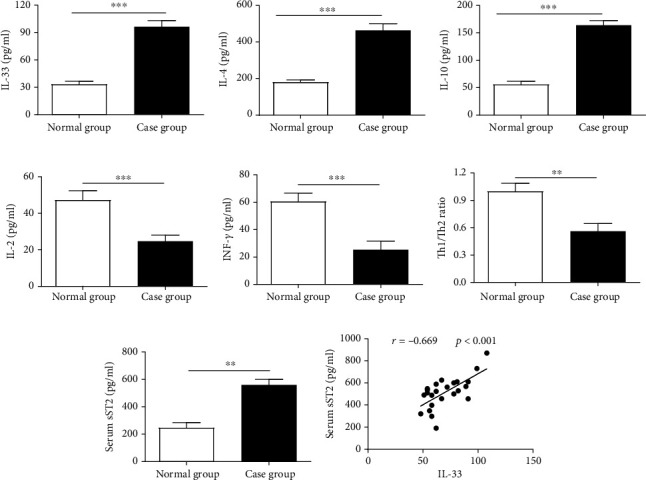
IL-33 expression is elevated, and Th1/Th2 ratio is imbalance in infants with acute bronchiolitis. (a) ELISA assay was used to detect the production of IL-33 in serum. (b, c) The levels of Th2-related cytokines IL-4 and IL-10 were examined by ELISA assay. (d, e) The levels of Th1-related cytokines IL-2 and INF-*γ* were examined by ELISA assay. (f) Cell flow cytometry was used to detect the ratio of Th1/Th2 cells. (g) ELISA assay was used to detect the production of ST2 in serum. (h) Correlation analysis is used to calculate the correlation between IL-33 and ST2 in serum. All data were expressed as the mean ± SD (*n* = 3). Each value of ^∗^*p* < 0.05, ^∗∗^*p* < 0.01, and ^∗∗∗^*p* < 0.001 was deem to have significant differences.

It was reported that the production of Th2 cytokines IL-4 and IL-10 was promoted by IL-33 [18], and Th2 immune response was described to influence the pathogenesis of respiratory syncytial virus (RSV) acute bronchiolitis [19]. Firstly, the expression of IL-33 in serum of acute bronchiolitis infants infected by RSV was examined by ELISA assay; we found that IL-33 concentrations in AVB infants were significantly increased compared to normal subjects (Figure 1(a)). Acute bronchiolitis elicited increased levels of IL-4 and IL-10 (Figures 1(b) and 1(c)) but decreased level of IFN-*γ* and IL-2 (Figures 1(d) and 1(e)). We also examined that the ratio of Th1 and Th2 cell was lowered in peripheral blood of acute bronchiolitis than normal infants by flow cytometry (Figure 1(f)). In addition, infants with acute bronchiolitis had a 2.87-fold higher level of serum (sST2) than those normal cases (Figure 1(g)). The ST2 protein expression was positively correlated (*r* = 0.669, *p* < 0.001) with IL-33 production in acute bronchiolitis cases (Figure 1(h)).

### 3.2. NF-*κ*B/IL-33/ST2 Axis Is Mediated the Th2 Cytokine Levels and BAL Cell Number Induced by RSV

**Figure 2:**
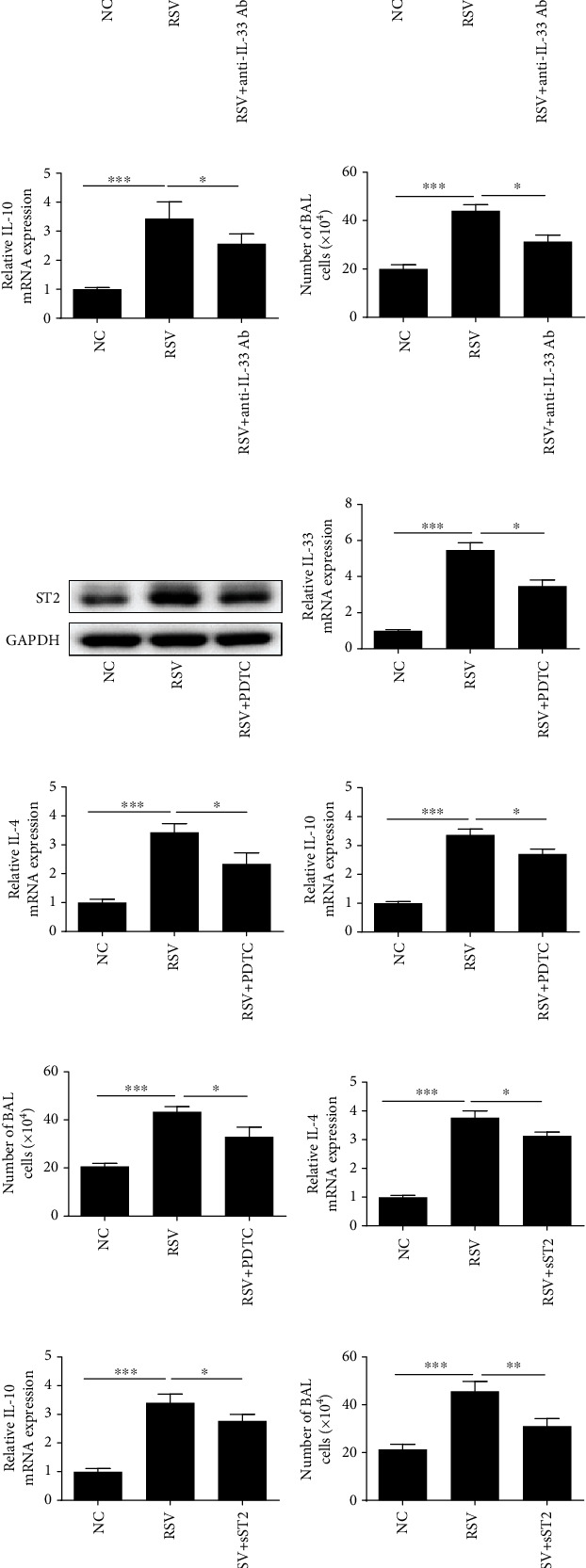
NF-*κ*B/IL-33/ST2 axis is mediated the Th2 cytokine levels and BAL cell number induced by RSV. (a) Western blot analysis was performed to examine the abundance of p-I*κ*Ba, ST2, p-p65, and p-p50. (b, c) RT-PCR analysis was used to examine the mRNA expression of Th2-related cytokines IL-4 and IL-10 after IL-33 antibody treatment. (d) Inflammatory cell number of bronchoalveolar lavage fluid after IL-33 antibody treatment. (e) ST2 protein abundance was examined by western blot analysis. (f) The IL-33 mRNA expression in lung tissues was detected by RT-PCR. (g, h) RT-PCR analysis was used to examine the mRNA expression of Th2-related cytokines IL-4 and IL-10 after PDTC treatment. (i) Inflammatory cell number of bronchoalveolar lavage fluid after PDTC treatment. (j, k) PCR analysis was used to examine the mRNA expression of Th2-related cytokines IL-4 and IL-10 after sST2 treatment. (l) Inflammatory cell number of bronchoalveolar lavage fluid after sST2 treatment. All data were expressed as the mean ± SD (*n* = 3). Each value of ^∗^*p* < 0.05, ^∗∗^*p* < 0.01, and ^∗∗∗^*p* < 0.001 was considered to be significant differences.

Previous studies have shown that NF-*κ*B is involved in the process of RSV-induced acute bronchiolitis [20], and NF-*κ*B inhibitor dimethyl fumarate inhibited IL-33 production [21], but its specific mechanism is unclear. Moreover, sST2 is another mode of existence of ST2. It is reported that sST2 can competently bind with IL-33, interfere with the coupling of IL-33 and ST2, and then inhibit the biological effects of IL-33. To confirm whether the level of Th2 cytokines IL-4 and IL-10 was mediated by IL-33, we used anti-IL-33 antibody by intranasal injection 30 *μ*g/mouse 2 h before challenge with RSV on 3 consecutive days (14, 15, and 16). Western blot analysis showed that abundances of ST2, p-I*κ*Ba, p-p65, and p-p50 were all induced by RSV, and ST2 abundance was markedly inhibited after treated with anti-IL-33 antibody prior to expose to RSV. However, the abundance of I*κ*Ba, p65, and p50 was not significantly decreased in the anti-IL-33+ RSV groups (Figure 2(a)). The mRNA expression of Th2 cytokines IL-4 and IL-10 elevated by RSV in lung tissues was significantly decreased in the anti-IL-33+ RSV groups (Figures 2(b) and 2(c)). Moreover, IL-33 antibody strongly suppressed the BAL cells activated by RSV (Figure 2(d)). In order to further verify whether there is a relationship between NF-*κ*B and IL-33, we used NF-*κ*B specific inhibitors (PDTC) to inject the mice prior 2 h to activate by RSV. As shown in Figures 2(e) and 2(f), the protein abundances of the ST2 and mRNA expression of IL-33 in mouse lung tissues induced by RSV were elevated and were significantly inhibited in the PDTC+RSV groups. Consistent with the role of IL-33 antibody, the mRNA expression of Th2 cytokines IL-4 and IL-10 elevated by RSV in lung tissues was also significantly inhibited in the PDTC+ RSV groups (Figures 2(g) and 2(h)). Besides, PDTC treatment strongly suppressed the BAL cells activated by RSV (Figure 2(i)). Furthermore, the levels of IL-4 and IL-10 (Figures 2(j) and 2(k)), and BAL cells (Figure 2(l)) elevated in the RSV groups were significantly inhibited in the RSV + sST2 groups. However, the abundance of IL-33 and NF-*κ*B (p-p50, p-p65, and p-I*κ*Ba) increased in the RSV groups was not suppressed in the sST2 + RSV groups.

### 3.3. Acute Bronchiolitis Induced by RSV in a Mouse Model Is Attenuated after Inhibition of NF-*κ*B/IL-33/ST2 Pathway

**Figure 3:**
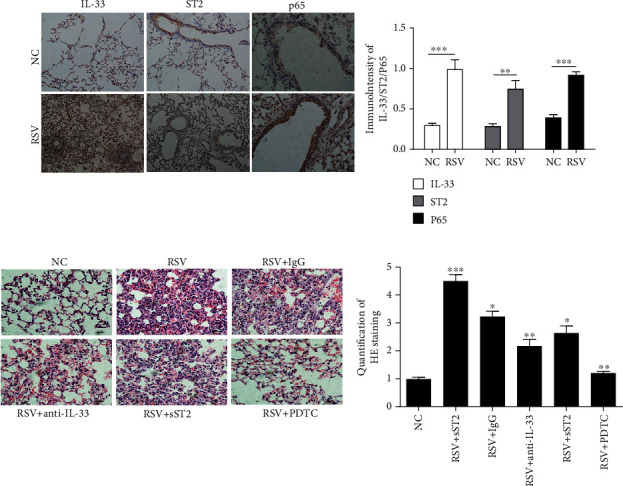
RSV-induced mouse model is attenuated after inhibition of NF-*κ*B/IL-33/ST2 pathway. (a) Immunohistochemical analysis of IL-33, ST2, and p65 abundance in saline-exposed control mice (NC); RSV-treated mice (RSV) was performed (original magnification ×400, scale bar 100 *μ*m) and scored (right graph). (b) H&E staining of lung tissue in different group mice was analyzed by Image-proplus 6.0 (original magnification ×400). The data are presented as mean ± SEM and were analyzed by Student's *t*-test (NC group, *n* = 8; RSV group, *n* = 7; RSV + IgG = 6; RSV + IL − 33 antibody = 6; RSV + PDTC = 7; RSV + sST2 = 8). Each value of ^∗^*p* < 0.05, ^∗∗^*p* < 0.01, and ^∗∗∗^*p* < 0.001 was considered to be significant differences.

To further confirm the role of NF-*κ*B/IL-33/ST2 pathway in RSV-induced AVB in a mouse model, immunohistochemical staining was performed in this study. Representative IL-33, ST2, and p65 immunostaining was examined in saline-exposed control mice (NC) and RSV-treated mice (RSV). Immunostaining results showed that the abundances of IL-33, ST2, and p65 were all increased in acute bronchiolitis induced by RSV than NC groups (Figure 3(a)). In addition, HE staining assay indicated that RSV significantly thickened the trachea wall, widened intercellular space, and enhanced inflammatory cell infiltration which was attenuated by anti-IL-13 antibody, PDTC, and sST2 treatment, representatively (Figure 3(b)).

### 3.4. Macrophages Are Important Sources of IL-33 and Are Regulated by NF-*κ*B Pathway in RSV-Induced Mice

**Figure 4:**
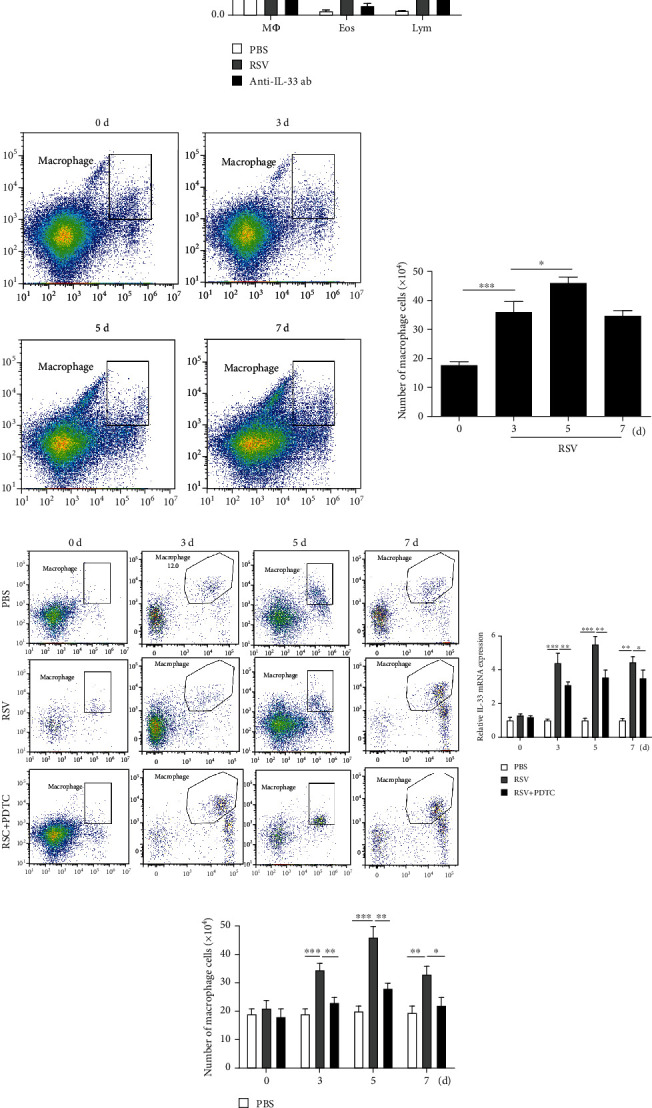
Macrophages are important sources of IL-33 and are regulated by NF-*κ*B pathway in RSV-induced mice. (a) The number of alveolar macrophages, lymphocyte, and eosinophils in BAL of mice. (b) Flow cytometry analysis was performed to sort alveolar macrophages at different times after RSV infection. (c) Flow cytometry analysis was performed to sort alveolar macrophages at different times after RSV + PDTC treatment. (d) The IL-33 expression in alveolar macrophages at different times after RSV + PDTC treatment was examined by RT-PCR analysis. All data were expressed as the mean ± SD (*n* = 3). Each value of ^∗^*p* < 0.05, ^∗∗^*p* < 0.01, and ^∗∗∗^*p* < 0.001 was considered to be significant differences.

To further clarify the main cellular source of increased IL-33 secretion after RSV infection, inflammatory cells in BAL were classified and counted. We found that the number of alveolar macrophages and eosinophils in BAL mice increased significantly after RSV infection (Figure 4(a)); flow cytometry analysis showed that the number of macrophages was the largest and reached the peak on the fifth day after RSV infection (Figure 4(b)). We further verified whether NF-*κ*B pathway was involved in RSV-induced macrophage number and IL-33 production in macrophages; we found that both the number of macrophages and expression of IL-33 increased in the RSV groups were reduced in the PDTC+RSV groups (Figures 4(c) and 4(d)).

### 3.5. RSV-Induced IL-33 Expression Was Inhibited through NF-*κ*B Pathway In Vitro

**Figure 5:**
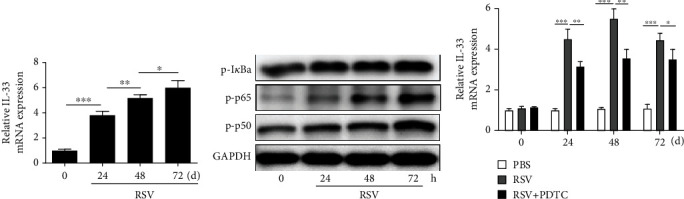
RSV-induced IL-33 expression was inhibited through NF-*κ*B pathway in vitro. (a) RT-PCR analysis was performed to examine the IL-33 expression in RAW264.7 cells at different time after RSV-induced. (b) Western blot analysis was performed to examine the abundance of I*κ*Ba, p65, and p50. (c) The IL-33 expression in macrophages at different times after RSV + PDTC treatment was examined by RT-PCR analysis. All data were expressed as the mean ± SD (*n* = 3). Each value of ^∗^*p* < 0.05, ^∗∗^*p* < 0.01, and ^∗∗∗^*p* < 0.001 was considered to be significant differences.

To further validate the role of NF-*κ*B pathway on the production of IL-33 in RSV infected macrophages, RAW264.7 macrophage line was used in this study. RT-PCR analysis showed that the IL-33 expression infected by RSV was markedly elevated after 24 h and at a time-dependent manner (Figure 5(a)). Consistent with the above results, the protein abundance of I*κ*Ba, p65, and p50 was also increased at a time-dependent manner in RSV-induced macrophages (Figure 5(b)). Furthermore, the expression of IL-33 increased in RSV activated RAW264.7 cells was significantly inhibited by PDTC (Figure 5(c)).

## 4. Discussion

Respiratory syncytial virus infection can aggravate airway inflammation and promote the occurrence and development of asthma [22]. If effective treatment measures are not taken in time, children with acute bronchiolitis may suffer from repeated wheezing and develop asthma in the future, which seriously affects pulmonary function and brings heavy mental and economic burden to their families and society [23].

IL-33 can promote helminth infection and alleviate atherosclerosis by promoting Th2 immune response [11]. In the process of airway inflammation induced by RSV, IL-33 and its specific receptor of ST2 increased significantly [24]. However, the role of IL-33/ST2 pathway on RSV-induced acute bronchiolitis and its molecular mechanism is unknown. In our study, we demonstrated that the levels of IL-33 and Th2-related cytokines IL-4 and IL-10 in RSV infected acute bronchiolitis in serum of infants were significantly increased. Besides, the Th1-associated cytokines IL-2 and INF-*γ* and Th1/Th2 cell ratio were markedly reduced in infant serum of acute bronchiolitis. Furthermore, RSV-induced Th2-related cytokines IL-4 and IL-10 in lung tissues were decreased by IL-33 antibody treatment. In addition, we also found that the increase of inflammatory cells in lung tissue infected by RSV was inhibited by IL-33 antibody. These data indicated that IL-33 plays an important role in acute bronchiolitis infants infected by RSV.

ST2 is one of the receptors of IL-1 family and widely expressed in many kinds of cells, especially mast cells and helper T cells [25]. Without proinflammatory stimulation, IL-33 only exists in the nucleus of inflammation and immune cells. In many disease processes, activated and released IL-33 can play an important role by combining with ST2 [26, 27]. In addition, soluble ST2 (sST2) as a bait receptor of IL-33 can directly bind to IL-33 and inhibit the biological function of IL-33 [28, 29]. In this study, we confirmed that the ST2 gene expression was positively correlated with IL-33 production in acute bronchiolitis cases. Both IL-33 antibody and sST2 could reversed RSV-induced Th2-related cytokine and pulmonary inflammatory damage. In addition, ST2 abundance can be inhibited in vivo and in vitro by IL-33 antibody treatment. Our results suggested that IL-33/ST2 pathway is mediated the RSV-induced acute bronchiolitis and maybe the potential targets for the prevention of asthma after acute bronchiolitis in infants.

Many kinds of cells can secrete IL-33 after stimulating inflammation. In addition to nonimmune cells such as epithelial cells and fibroblasts, macrophages, dendritic cells, and mast cells can also secrete IL-33 after stimulation [30]. In recent years, increasing evidences indicate that innate immune cells may be an important source of IL-33 in the process of respiratory viral infection [30]. By flow cytometry analysis, we found that inflammatory cells increased significantly after RSV infection in mice, mainly macrophages, and the IL-33 expression in macrophages was elevated markedly. In addition, we also found that IL-33 antibody could reduce the number of macrophages induced by RSV. These results indicated that RSV increases the number of macrophages and leads to the increase of IL-33 secretion; IL-33 could also further promote the increase of macrophages.

Although evidence showed that IL-33 plays a biological role through NF-*κ*B pathway [20], IL-33 antibody treatment did not inhibit the expression of NF-pathway induce by RSV in our study, so we speculate that the NF-*κ*B pathway is not mediated by the inhibitory effect of IL-33 antibody on RSV-induced lung damage. Interestingly, we found that NF-*κ*B inhibitors can reduce RSV-induced lung damage in mice, and we also found that NF-*κ*B inhibitors can reduce the abundance of IL-33 in vivo and in vitro. This finding indicated that the NF-*κ*B pathway is mainly involved in the process of RSV-induced IL-33 secretion in mice.

In conclusion, we confirmed that NF-*κ*B/IL-33/ST2 axis is mediated the acute bronchiolitis by RSV infected. Our findings not only demonstrate the potential role of IL-33 antibody in attenuating RSV-induced lung damage but also provide a new insight into better prevention of RSV-induced asthma by mediating NF-*κ*B/IL-33/ST2 axis.

## Figures and Tables

**Table 1:** Sequences of primers for PCR.

Genes	Forward primer	Reverse primer
IL-33	5′-GTCGCCCTGGTACCAGTCCAG-3′	5′-AGGCCTGGCCCGAGTTGTCAG-3′
IL-4	5′-GGCCCGCTATTTGTTTGGTCA-3′	5′-GCTCCTCCCTTGCTTACCAG-3′
IL-10	5′-TAGACGCGCTGGGCGACAG-3′	5′-GTCGCCCCCTAACGCCGTAA-3′
GAPDH	5′-TTCGAAGCACCGGTCCC-3′	5′-TCTCAAGAGCAGCTCCAGT-3′

## Data Availability

All data collection and analysis were conducted under double-blind and supported by The Second People's Hospital of Changzhou, Affiliated Hospital of Nanjing Medical University.

## Supplementary Materials

Supplementary Materials Uncropped western blotting image.
