# Supplementary figures and images for: Inhibition of NF-κB/IL-33/ST2 Axis Ameliorates Acute Bronchiolitis Induced by Respiratory Syncytial Virus
Source: J Immunol Res. 2021 Aug 4;2021:6625551. doi: 10.1155/2021/6625551 (PMC8357524; doi:10.1155/2021/6625551)

wb original data

Figure 2A

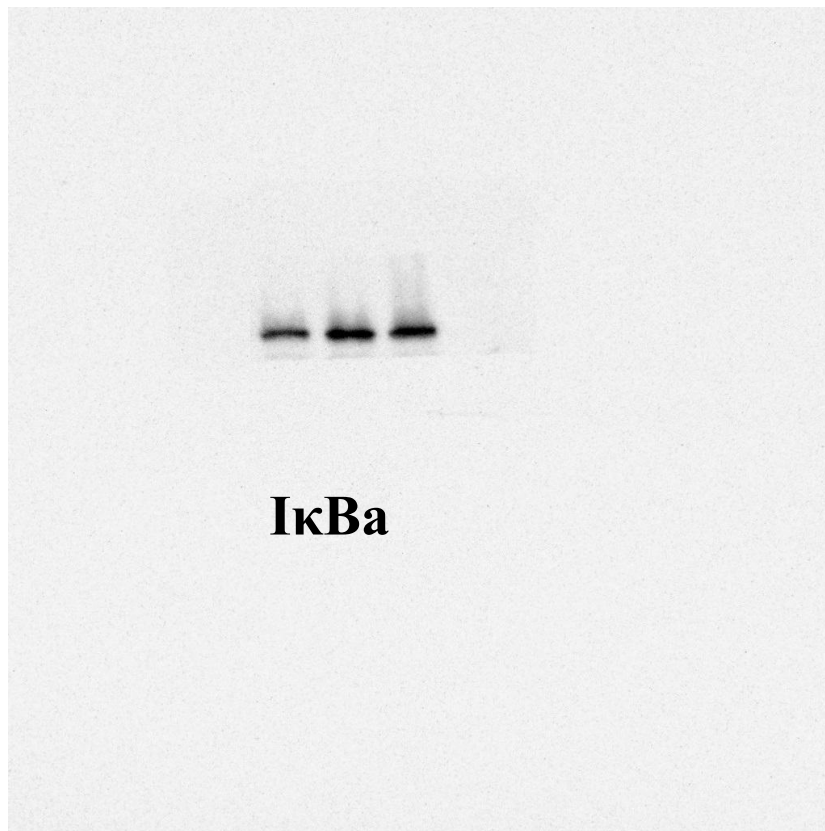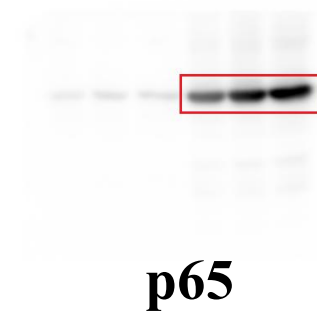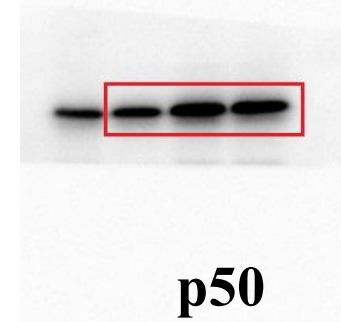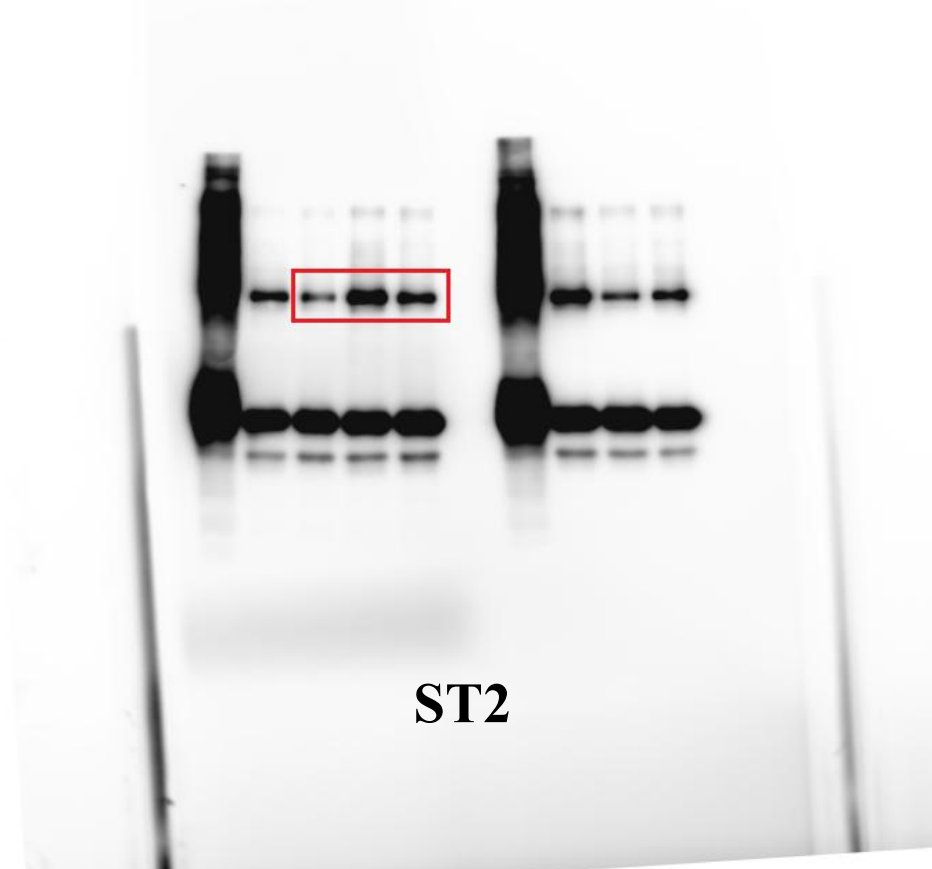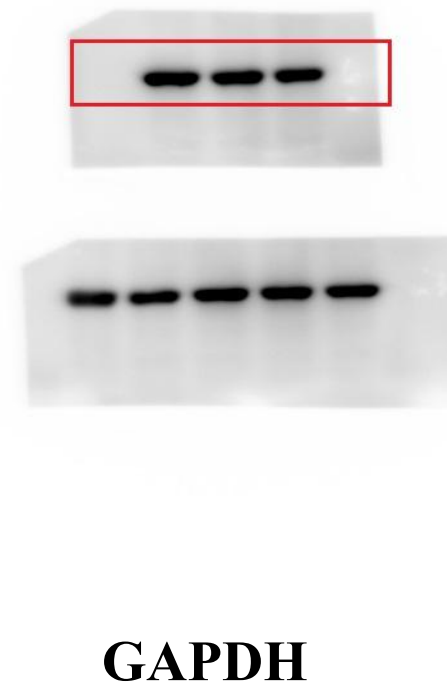

Figure 2E

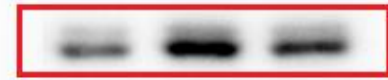

**ST2**

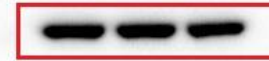

**GAPDH**

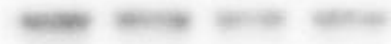

wb original data

Figure 5B

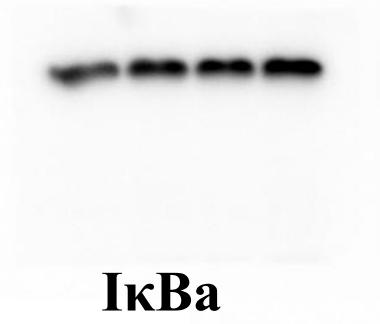

I $\kappa$ B $\alpha$

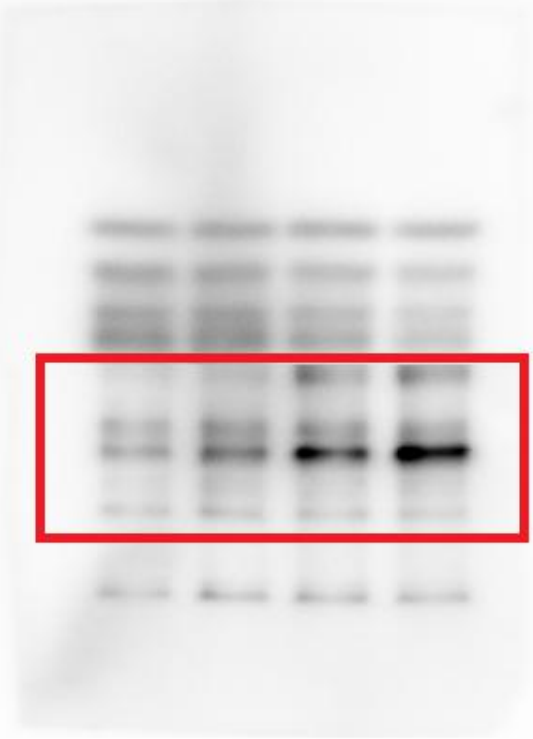

p65

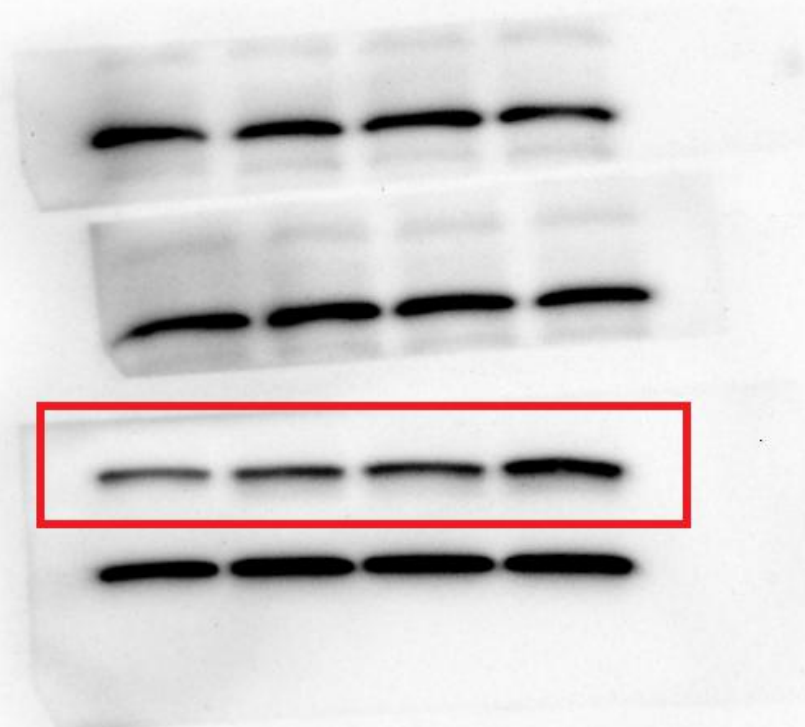

p50

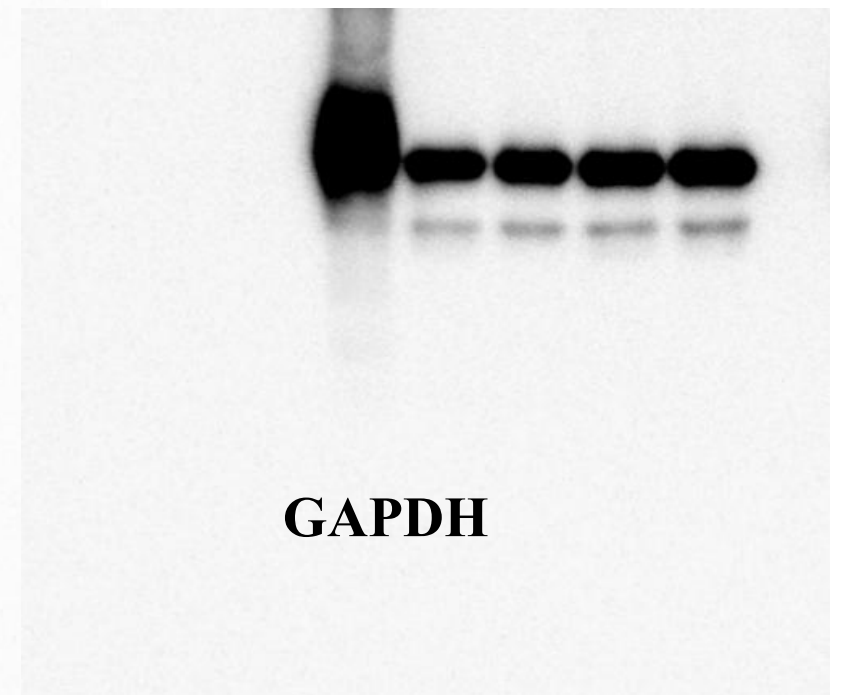

GAPDH

Supplement: Supplementary Materials — Uncropped western blotting image. [file 6625551.f1.pdf]
